# Systematic review on treatment of juvenile idiopathic arthritis – associated uveitis

**DOI:** 10.1186/1546-0096-12-S1-I6

**Published:** 2014-09-17

**Authors:** Arnd Heiligenhaus

**Affiliations:** 1 Ophthalmology, St Franziskus Hospital, Muenster, Germany

## Background

Juvenile idiopathic arthritis (JIA) is commonly complicated by chronic uveitis that frequently leads to visual loss.

## Methods

Review of the current literature on the treatment of JIA - associated uveitis.

## Results

Therapy of JIA-associated uveitis is guided by the severity of inflammation and complications. Topical corticosteroids are generally used as the initial treatment. Severe uveitis is commonly treated with immunosuppressive drugs. Methotrexate is presently the first-choice agent. If uveitis is not responding, another immunosuppressive agent or biological is applied. Currently, adalimumab is the preferred TNF-inhibitor. In refractory disease, other biologicals are used (e.g., rituximab, tocilizumab or abatacept). Ocular corticosteroid injections / - implantations are considered as “rescue therapy”.

## Conclusions

Controlled studies are warranted to offer most effective and safe therapy for children with JIA - associated uveitis. Better knowledge of the basic mechanisms underlying the disease and of the molecules that are important for regulating inflammation may help to create new and more specific treatment approaches, and to improve disease monitoring.

## Disclosure of interest

None declared.

